# MLTreeMap - accurate Maximum Likelihood placement of environmental DNA sequences into taxonomic and functional reference phylogenies

**DOI:** 10.1186/1471-2164-11-461

**Published:** 2010-08-05

**Authors:** Manuel Stark, Simon A Berger, Alexandros Stamatakis, Christian von Mering

**Affiliations:** 1 Institute of Molecular Life Sciences and Swiss Institute of Bioinformatics, University of Zurich, Switzerland; 2 Ph.D. program in Molecular Life Sciences, University of Zurich and Federal Institute of Technology (ETH), Zurich, Switzerland; 3 The Exelixis Lab, Department of Computer Science, Technische Universität München, Germany

## Abstract

**Background:**

Shotgun sequencing of environmental DNA is an essential technique for characterizing uncultivated microbes *in situ*. However, the taxonomic and functional assignment of the obtained sequence fragments remains a pressing problem.

**Results:**

Existing algorithms are largely optimized for speed and coverage; in contrast, we present here a software framework that focuses on a restricted set of informative gene families, using Maximum Likelihood to assign these with the best possible accuracy. This framework ('MLTreeMap'; http://mltreemap.org/) uses raw nucleotide sequences as input, and includes hand-curated, extensible reference information.

**Conclusions:**

We discuss how we validated our pipeline using complete genomes as well as simulated and actual environmental sequences.

## Background

In the field of microbial genomics, successful laboratory cultivation of naturally occurring microbes has become a major bottleneck [1-3]; this limits and biases our understanding of the biochemical capabilities and ecological roles of microbes in their habitats. Since cultivation is a prerequisite for standard genome sequencing approaches, we are still lacking genomic information for many important microbial lineages (including entire phylum-level groups [4,5]). In addition, there is a sequencing backlog even for those strains that have been cultivated successfully; this however is being addressed now by directed sequencing efforts that are underway [6,7]. Nevertheless, the severe biases and the large gaps in the worldwide collection of cultivated isolates make it difficult to fully appreciate evolutionary processes and microbial ecology, or to exploit the large repertoire of microbial genes that might be relevant to medicine and biotechnology. While techniques that analyze single cells, such as multiplexed microfluidics PCR [8] or single-cell genome sequencing [9,10], can provide unequivocal genomic data in the absence of cultivation, these methods are still limited in terms of throughput and usability. Thus, the approach that presently generates the largest amount of unbiased microbial genome sequence data is 'metagenomics' ([11]; also termed 'environmental sequencing').

More than 200 metagenomics projects are currently registered [5] at various stages of completion; these address a wide variety of habitats and microbial lifestyles [12-16]. Typically, in such projects, an environmental sample is processed by lysing cells and indiscriminately isolating genomic DNA; the latter is then fragmented and shotgun-sequenced to a desired depth. However, even when employing the latest next-generation, high-throughput DNA sequencing technologies, the large complexity and genomic heterogeneity of natural microbial communities often preclude *de novo *assembly of complete genomes from the data - instead, a large number of short to medium-sized sequence fragments are obtained. From these, quantitative inferences can already be made regarding genome sizes [17,18], recombination rates [19], and functional repertoires [20,21], among others. However, many of the perhaps more important ecological questions require the assignment of the sequence fragments to the microbial lineage they originate from, a process called 'binning' [12,22].

An increasing number of algorithms have been devised for this task; these can largely be divided into two groups. The first consists of 'unsupervised' approaches [23-27], in which sequences are binned using signature-based algorithms that focus on nucleotide compositional signals (reflected in the relative frequencies of short nucleotide 'words'). These approaches require no external reference information *a priori*; instead, they learn to distinguish the major taxonomic groups from the data itself (although subsequent assignment to known taxonomic entities is often done). In contrast, 'supervised' approaches [28-34] require extensive, annotated, external reference information. For the most part, these approaches interpret the results of large-scale homology searches against sequence databases, sometimes followed by phylogeny reconstruction; the external reference information is usually derived from the available fully sequenced microbial genomes. For both types of approaches, the various implementations differ greatly in their speed, accuracy, coverage, ease of installation and use, and in the interpretation and visualization of the results. Owing to the size and nature of the input data, formal phylogenetics algorithms are relatively rarely used in these pipelines, with three exceptions: Maximum Parsimony in [33], Neighbor Joining in [29], and an approximate Maximum Likelihood approach in [34]. That the Maximum Likelihood approach has not been applied more frequently is somewhat surprising, since it is arguably among the most accurate and best-described techniques in phylogenetics [35-38]. One reason for this is presumably the high computational cost of this approach, which makes it difficult to execute for very large numbers of sequence fragments.

Here, we describe a software framework ("MLTreeMap") that does employ full Maximum Likelihood, and which is specifically designed for metagenomics sequences. We significantly reduced the computational costs through algorithmic improvements, as well as through a focus on a restricted (but user-extensible) set of informative gene families. The aim of the framework is to cover the high-accuracy end of the tool spectrum, with a particular focus on consistency across different sources of input data. To achieve this, the package, a) starts from raw nucleotide sequences to avoid inconsistencies arising from different gene-calling strategies, b) corrects for frame-shifts and other errors on the fly to optimally extract marker genes, c) includes searches against 'off-target' reference sequences to avoid the detection of undesired deep paralogs, d) concatenates marker genes when several of them are observed in a given sequence fragment, and e) offers intuitive visualization features, both via the command-line as well as via the web-server. The framework contains hand-curated reference phylogenies and alignments; in the first full release that we describe here (MLTreeMap version 2.011), these references encompass a total of 44 distinct gene families that have been selected to address both taxonomic as well as functional aspects of microbial assemblages.

## Results and Discussion

**Figure 1:**
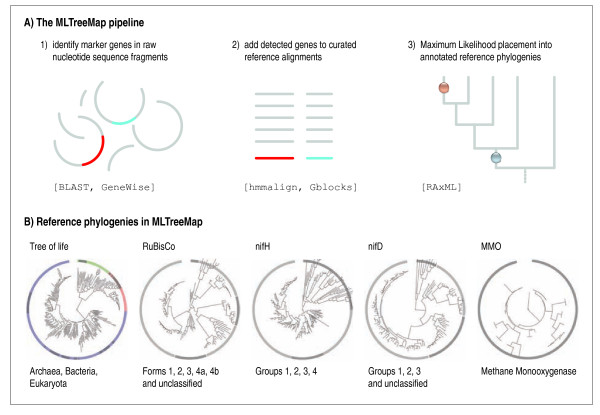
**MLTreeMap: Placing anonymous sequence fragments into reference phylogenies**. Top: overview of the procedure. Informative marker genes (or fragments thereof) are automatically extracted from raw, un-annotated nucleotide sequence fragments, aligned to reference sequences and then placed into externally provided gene trees using RAxML. Below: Overview of reference phylogenies that are currently available in MLTreeMap.

We have previously outlined [31] and used [39,40] a preliminary version of the MLTreeMap pipeline; however, this initial implementation was not designed for deployment, only focused on phylogenetic information, and was computationally very inefficient (it required up to several hours of CPU time to assign a single nucleotide sequence fragment). We have since achieved a more than 100-fold speed-up, mainly by using more efficient pipeline code, and by switching the employed Maximum Likelihood phylogenetics engine from TREE-PUZZLE [41] to RAxML [42,43]. This switch also enabled us to deploy recent optimizations inside RAxML that were specifically devised for this purpose [Berger et al., submitted; preprint available at http://arxiv.org/abs/0911.2852v1]. The basic work-flow of a fully automated MLTreeMap run proceeds as follows (Figure 1): First, a batch of input sequences (i.e., un-annotated nucleotide sequences) are searched for the presence of marker genes, by running BLASTX against a curated collection of reference proteins (including 'off-target' proteins where necessary). In a next step, all detected instances of these marker genes are extracted using GeneWise [44], based on Hidden Markov Models (HMMs) that are provided as part of the MLTreeMap pipeline; this establishes protein-coding open reading frames and exhibits some tolerance to sequencing errors such as frame-shifts or gaps. The query proteins are then aligned to the corresponding reference proteins using hmmalign [45], and the resulting alignments are concatenated in case more than one marker gene is located on a given fragment (this latter step only applies to phylogenetic markers). Next, alignments are subjected to mild gap-removal [46]; and subsequently they are submitted to RAxML. There, the sequences are placed in their most likely position within the corresponding reference phylogeny. Importantly, RAxML is instructed to fully maintain the input topology of the reference phylogeny and to keep it fixed during the computations. Upon launching, RAxML initially optimizes the Maximum Likelihood model parameters and computes all branch-lengths of the reference tree, based on the alignment provided. Next, RAxML will insert (and subsequently remove again) the query sequence(s) one at a time into every possible branch of the reference tree, re-optimizing the three branch lengths at the insertion position for each attempt. The best-scoring position (branch) for each query sequence is then reported. Optionally, RAxML can use non-parametric bootstrap to account for placement uncertainty. For the bootstrap replicates, heuristics are deployed that only assess the top 10% most promising placement branches as computed on the original (non-bootstrapped) alignment and thereby reduce run times for bootstrap placements by one order of magnitude. Note that, under the settings chosen for MLTreeMap, the actual likelihood computations in RAxML follow the standard Maximum Likelihood approach under a standard protein evolution model, for maximum accuracy. Finally, the results are aggregated, reported in human-readable form and visualized graphically in the context of the reference trees (Figure 1). Currently, 40 of the reference protein families that we provide are collectively used to assess the taxonomic composition of the input sequences (these 40 families were selected based on universal occurrence in all three domains of life, as near-perfect single-copy genes [47]). Another four families serve as indicators for the presence of crucial metabolic pathways (nitrogen fixation, photosynthesis and methane assimilation). In the current implementation, the processing of an amount of DNA sequences that is equivalent to an average microbial genome takes about three to four hours on a single CPU (more when bootstrapping is requested; for example, the above runtime changes to 7 hours when 10 bootstraps are done in each RAxML run). The performance scales roughly linearly with the amount of DNA to be processed; for example, a medium sized metagenome (C1-oxidisers in lake water [48], at 37 Mb) requires about 30 hours to compute on a single CPU; a larger metagenome (220 Mb from a hot spring) requires close to 200 hours. Since the individual DNA fragments can be assessed independently, the pipeline can seamlessly be deployed onto a compute cluster (by splitting the input, and aggregating the results afterwards).

**Figure 2:**
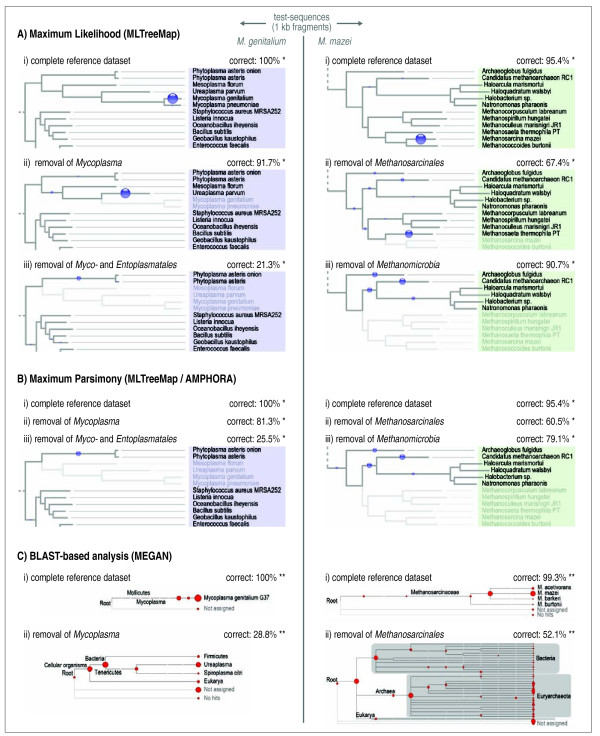
**Leave-one-out validation: examples**. Individual query genomes were fragmented (1'000 bp fragments) and then placed into reference trees from which the corresponding genomes (or entire clades) had been removed. The assignments are shown graphically (small circles). Note how the placements become increasingly scattered and imprecise upon removal of increasingly deep reference information. MLTreeMap is shown compared to two popular approaches (note that MEGAN, while the least accurate, applies to a much larger fraction of reads in a given sample and thus achieves the best coverage). Definitions of test success: *assignments are designated as correct when they are no more than two nodes away from the target position in the tree. **for MEGAN, assignments are designated as correct when they are mapping to the target phylum.

To validate the performance of the MLTreeMap pipeline, we first tested its accuracy on short sequences of known origin. These were generated by artificially fragmenting fully sequenced genomes into non-overlapping stretches of 1'000 base pairs each (this length corresponds to current read lengths of the Sanger sequencing technology, and it also matches the projected length of the upcoming next release of the 454 pyrosequencing technology). To avoid circularity, we removed the corresponding genomes from our reference alignments and pruned them from the trees. Thus, our testing amounts to leave-one-out cross-validation. Note that our phylogenetic reference tree is already non-redundant at the genus level (with a few exceptions), meaning that removal of the query genome usually results in the next best relative to be available only at the phylogenetic rank of 'family' or higher. The performance of our approach was compared to that of two widely used, previously published approaches, MEGAN [28] and AMPHORA [33], which are based on BLAST searches or Maximum Parsimony insertions, respectively. The algorithmic challenge of our test varies from query genome to query genome, depending on its phylogenetic position (depth) in the reference phylogeny. This is illustrated, for two exemplary genomes, in Figure 2: all three approaches deliver a good accuracy when the query genome remains in the reference (i.e., 95% to 100% of correct placements, see top of Figure 2). However, when removing the query genome from the reference, together with increasingly distant relatives, the accuracy of all three approaches decreases, as expected. This is relevant, because actual environmental sequence fragments will often be fairly unrelated to any fully sequenced genome. Since in our test each query genome is represented by 40 independent reference genes, the resulting placements are spread out over the tree; this is a good visual indication of the nature and extent of the placement error (Figure 2). For the two arbitrary genomes that we chose as examples in Figure 2, Maximum Likelihood and Maximum Parsimony were both performing significantly better than the BLAST-based heuristics implemented in MEGAN. Between the two, Maximum Likelihood performed better in three instances, whereas Parsimony insertion performed better in one instance (note that all pre-processing steps and reference sequences were kept exactly the same for the latter two approaches, in order to facilitate their direct comparison).

**Figure 3:**
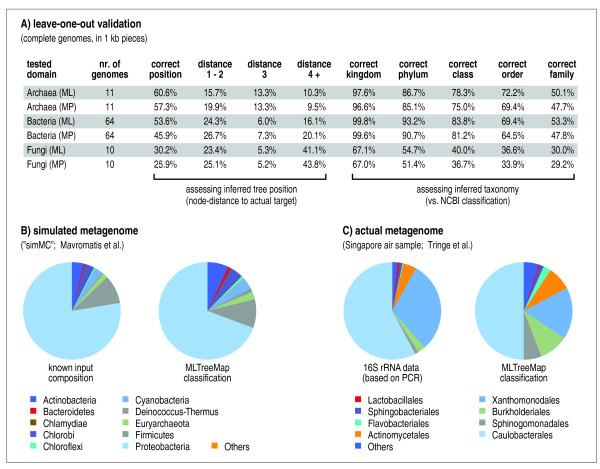
**Systematic validation**. MLTreeMap is tested on three different types of input (fragmented genomes, as well as simulated and real metagenomes). In all cases, the pipeline has been run with default settings, using the extended reference phylogeny based on Ciccarelli et al. [47].

We next performed this test systematically, based on 85 complete genomes (11 Archaea, 64 Bacteria and 10 single-celled Eukaryotes (fungi); see Figure 3). This involved testing 406'900 sequence fragments, of which 4'186 were found to contain at least one of our phylogenetic marker genes (i.e., our pipeline typically addresses only about 1% of the sequences in any given sample, by focusing on the most informative parts). We observed that, overall, Maximum Likelihood placed 47.2% of the query sequences at precisely the correct position in the tree, and another 21.3% in close vicinity (i.e., at most two nodes away in the tree). This compares favorably to Maximum Parsimony insertion, using the exact same sequence input (44.8% and 22.0%, respectively). This can also be described in taxonomic terms: Maximum Likelihood places 86.0% of the query sequences within the correct phylum, and 61.2% even within the correct order; these numbers are 83.8% and 55.6% for Maximum Parsimony, respectively. The gain in accuracy over Maximum Parsimony is not dramatic, but it is statistically significant: when re-testing the fragmented bacterial genomes in 1000 bootstrap runs (i.e., randomly sampling genome fragments with replacement), the distributions of accuracy scores for the two approaches were at least four standard deviations apart - testing each of the levels 'phylum', 'order' and 'family'. Overall, there are notable differences with respect to the three kingdoms of life: Bacteria are currently placed with the highest accuracy, with Archaea being a close second, whereas Eukaryotes are assigned with comparatively low accuracy. The difficulties with Eukaryotes can be partly attributed to the presence of more paralogs, and introns (the latter can fragment marker genes), but presumably also to mitochondria and other organelles, which introduce non-eukaryotic versions of the marker genes we employ.

**Figure 4:**
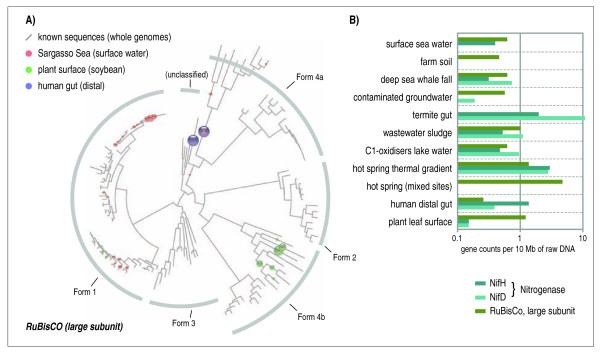
**Functional characterization of metagenomes**. A) Three published environmental sequence datasets have been searched for instances of the RuBisCo and RuBisCo-like enzyme families, using MLTreeMap. Colored spheres represent sequences mapping to a specific position in the tree, whereby the area of each sphere indicates the relative amount of sequences. The resulting placements are largely non-overlapping, suggesting distinct functional RuBisCo classes encountered/required at each of the environmental sites. B) Several datasets, as available at [69] and [70], were assessed with respect to two metabolic functions (CO^2 ^fixation, and nitrogen fixation, respectively). All counts were normalized with respect to sampling depth, and are thus directly comparable.

We also assessed our procedure by applying it to entire metagenomics datasets, both simulated [49] and real [50]. For the latter, independent taxonomic information is available, which is based on 16 S ribosomal RNA genes that have been PCR-amplified and sequenced from the very same sample [50]. As is summarized in Figure 4, the results for both datasets are in good quantitative agreement with the known (or measured) composition of the input data. In the case of the simulated dataset [49], the task is necessarily somewhat easier, since this set has been assembled by fragmenting known genomes, and many of these genomes are also contained in our reference phylogeny. Nevertheless, of the 113 genomes that contributed to the 'simMC' dataset [49], more than half (59) are not contained in our reference; and of these, 7 are not even represented at the genus level. In addition, the simulated set contains genomes at widely differing levels of sequence coverage, and the genome sizes are also quite variable (spanning almost one order of magnitude). In spite of this, the overall taxonomic composition is reliably recovered by MLTreeMap, and none of the phyla known to be present in the sample have been missed. For the real metagenomics dataset [50], the actual 'target' composition is not known with much certainty, since the PCR-based assessment that has been reported together with the sample could itself exhibit intrinsic quantitative error. Indeed, we observe that the MLTreeMap classification appears somewhat more 'balanced' than the PCR-based classification (see Figure 3C: the two most abundant groups make up 88% in the PCR data, but only 67% in the MLTreeMap data). This observation is of course not conclusive: the actual composition of the original sample could well be more biased than reflected in the metagenome. We do note that the distribution of 16 S genes in the metagenome (not PCR-amplified) agrees somewhat better with the MLTreeMap classification than with the PCR-amplified 16 S genes (data not shown), so the observed discrepancy might at least partially be due to the known amplification biases of PCR reactions on mixed templates [51-53], or due to biases in cloning efficiency [54].

Finally, we tested the MLTreeMap pipeline not only with respect to taxonomic assignment, but also with respect to the functional characterization of samples. Currently, the pipeline covers four important enzyme families (RuBisCO, Nitrogenase/NifD, Nitrogenase/NifH, and Methane Monooxygenase). These families are represented by hand-curated alignments, and visualized in the form of annotated protein trees. Future versions of MLTreeMap will extend this set in order to cover a significantly larger number of important diagnostic protein/enzyme families that are indicative of core functions (metabolic and otherwise [55-59]). Figure 4A shows a typical result of MLTreeMap for the functional classification of a set of environmental sequence samples. Three datasets are shown, that each contain representatives of the RuBisCO enzyme family (Ribulose-1,5-bisphosphate carboxylase oxygenase). The mere presence of these genes in the sample could also have been deduced from simple BLAST searches on the data; however, the summary shown in Figure 4A reveals crucial, additional information: first, the mapped sequences show a clear separation into distinct sub-families of RuBisCO. The surface seawater sample is dominated by subfamily #1, the plant surface sample by subfamily #4b, and the distal human gut by subfamily #4a and other unclassified parts of the tree (subfamilies are designated according to [60]). Second, the functional placements tend to corroborate the taxonomic assignments that MLTreeMaps reports for the same samples (not shown); this enables checks for consistency and/or unexpected horizontal transfers. And third, the placements can be seen to differ dramatically in their distance from the root, that is, in their evolutionary 'depth' with respect to previously known members of the family. For example, in the case of the surface seawater, virtually all sequences were very close to the tips of the tree, in other words closely related to known examples of RuBisCO (mainly from Cyanobacteria and alpha-Proteobacteria). In contrast, instances of RuBisCO-like proteins in the human gut were observed much closer to the root, i.e., at a greater evolutionary distance from previously known sequences and in non-canonical subfamilies. From this, it would be much harder to predict their functions, and it is indeed conceivable that they are *not *functioning in CO^2 ^fixation, but rather in other, possibly sulfur-related metabolic pathways (methionine salvage or yet other, uncharacterized pathways [60-62]). The standardization and ease of use provided by MLTreeMap allow for consistent, semi-quantitative analysis of the functional coding potential of entire collections of metagenomics samples - as an example, Figure 4B shows combined data for 11 distinct metagenomes. In this case, the coding capacities for nitrogen fixation and CO^2 ^fixation have been compared across samples and sites. Large differences become apparent, including the known paucity of nitrogen fixation genes in some environments [63], but also surprises such as nitrogenase-like genes in the distal human gut. Here again, the availability of the annotated reference trees in the MLTreeMap output is crucial: the sequences are likely of a non-canonical, archaeal type, related to genes in *Methanobrevibacter smithii*, and are thought to function in a process other than nitrogen fixation [64,65].

For both, functional as well as taxonomic assignments, MLTreeMap offers a number of user-definable parameter settings. Users can chose which of two phylogenetic reference trees to use (modified from [7] or [47]), and whether to use Maximum Likelihood or Maximum Parsimony (the latter works faster but is somewhat less accurate; see Figures 2 and 3). When choosing Maximum Likelihood, users can also request bootstrap replicates. However, bootstrapping will in most cases not be necessary since the input data is already divided into many independent sequence fragments (these constitute 'bootstraps' in some sense; the fragmentation is due to the lack of assembly in most metagenomics projects). Bootstrapping could of course be turned on for specific cases of interest, but for assessing entire datasets it is probably less advisable. This is because individual RAxML runs using all the columns of a given sequence alignment yield more accurate results than each individual bootstrapping run in which columns have been re-sampled [on average, only 65% of distinct input columns are used in each bootstrap, Berger et al., submitted; this becomes an issue particularly when input sequences are rather short to begin with]. The overall accuracy of MLTreeMap is fairly good already, but it could be further enhanced by improving the coverage and evenness of the reference trees and also by optionally giving deeply assembled contigs (i.e., those with high read coverage) correspondingly more weight in the final aggregation step. Future versions of the pipeline could also likely be optimized further with regards to computational speed - we note that currently much time is still spent outside RAxML, in the pre-processing steps. If further speed-ups can indeed be achieved, then the pipeline should cope well with further advances in sequencing technology - perhaps even to a point in the future when much of the raw data will be discarded immediately after sequencing, and only genes of interest (such as the phylogenetically and functionally informative genes assessed by MLTreeMap) will be kept.

## Conclusions

MLTreeMap performs consistent and rapid placements of metagenomics sequence fragments into high-quality, manually curated reference phylogenies - with high accuracy, albeit covering only a restricted fraction of any given sample (around 1%). It focuses on phylogenetically and functionally informative genes, thereby aiming to capture and characterize core aspects of a microbial community. MLTreeMap is one of only a few frameworks that can address microbial eukaryotes on an equal footing with prokaryotes, and it can easily be extended by the user (with any specific gene family of interest). The pipeline will likely be best put to use when analyzing hundreds of samples in comparison: this should ultimately reveal quantitative correlations between certain taxonomic clades and certain functional gene abundance profiles, thus helping to address the classic question of 'who does what' in microbial assemblages.

## Materials and methods

### Data Sources

Annotated protein-coding genes from fully sequenced genomes were downloaded from STRING [66] and RefSeq [67]. The phylogenetic 'tree-of-life' references were obtained from [7] and [47], but were subsequently modified: we removed genomes for which we were unable to obtain sequences, at the time, and added others. For the tree of [47], we made the representation of organisms non-redundant at the genus level, with a small number of exceptions for fast-evolving genera, and recomputed the best Maximum Likelihood tree, while keeping fixed the original topology of the published tree ('constraints' in RAxML). This computation was based on concatenated alignments of the exact same 40 reference genes as used by MLTreeMap. Note that the purpose of MLTreeMap is not to generate tree-of-life phylogenies *de novo*; instead these trees are provided externally [7,47], we therefore chose to maintain their published topology. For the four functional reference families, gene family information was obtained from KEGG [68] (*nifD: K02586, nifH: K02588, MMO: K08684*) and from STRING [66] (*RuBisCO: COG1850*). In total, the current release 2.01 of MLTreeMap contains 11,069 genes in the reference data; on average, each gene family of interest is represented by 252 genes.

### Implementation and Use

MLTreeMap is provided both online (albeit with input-size limitations) as well as offline in form of a command-line executable. The latter is designed with as few external runtime dependencies as possible: BLAST, GeneWise, HMMER and RAxML. Visualization of the results is optional, and a separate Perl-script (with additional dependencies) is provided for this purpose. When using the pipeline, individual reports are generated for each sequence fragment on which marker genes were detected. Aggregated reports are also generated, but this step may have to be repeated by the user (for example when running the pipeline in parallel on separate machines, or when re-weighting the fragments according to additional, external information such as assembly depth or sample size).

The MLTreeMap pipeline has only a few configurable parameters (including: choice of phylogenetic placement method, number of bootstraps, and choice of taxonomic reference phylogeny); other settings are hardcoded with the following default values: required significance of initial BLASTX hits (e = 0.01; database size fixed at 1'000'000), gap removal parameters for Gblocks (-t = p -s = y -u = n -p = t -b3 = 15 -b4 = 3 -b5 = h -b2 = [0.55 · #alignment_rows]), and required sequence length of the marker genes after alignment and gap removal (50 amino acids). Due to this latter threshold, the pipeline will not yield much useful information for samples with typical read lengths below 300 base pairs (indeed, 500 bp or longer is recommended). The Maximum Likelihood insertion in RAxML is typically done under the following settings: "-f v -m PROTGAMMAWAG" (the WAG substitution model yields the best likelihood scores on the phylogenetic reference trees, compared to all other amino acid substitution models available in RAxML; this was assessed using the RAxML "-f e" option for tree evaluation). For only 7 of the 44 protein families, a substitution model other than WAG is used (RTREV for COG0049, COG0090, COG0092, COG0093 and COG0100; CPREV for COG0201 and BLOSUM62 for Methane Monooxygenase). RAxML works with unrooted trees; however, the MLTreeMap pipeline reports all results in the context of rooted trees, for convenience (the re-rooting is hardcoded for each reference tree). Note that the actual Maximum Likelihood insertion step in MLTreeMap is clearly defined and fairly generic - it could in principle be performed also by software other than RAxML (for example by the PPLACER program; Matsen et al., personal communication; preprint at http://arxiv.org/abs/1003.5943). MLTreeMap can be compiled and executed locally, and previous versions are maintained at our website, for reference (together with the corresponding reference alignments and trees). We plan to update MLTreeMap yearly - each time updating the reference alignments with data from newly sequenced genomes, and extending the repertoire of functional reference families.

### Validation

For the validation tests based on whole genomes, the query genomes were artificially fragmented into non-overlapping, consecutive stretches of 1'000 base pairs each. Prior to each test, the respective genome was removed from the reference phylogeny to avoid circularity, and MLTreeMap placements were made using either Maximum Parsimony or Maximum Likelihood (all other settings were identical; bootstrapping was not used). The resulting placements were then compared to the known positions of the query genomes in the reference tree, either by assessing the node distance or the taxonomic assignment. For the latter, the newly placed fragment was assigned to the highest taxonomic rank for which all genomes in the clade below the placement branch were in agreement. For the tests based on simulated metagenomes, we chose the Phrap assembly of the 'medium complexity' simulated dataset, available at http://fames.jgi-psf.org/. The expected target composition of this set is not simply defined by the list of constituent genomes [49]; instead, since the relative genome representation depends on the read coverage of each genome in the simulated set, we weighted all genomes accordingly.

## List of Abbreviations

PCR: polymerase chain reaction; ML: Maximum Likelihood; MP: Maximum Parsimony; RuBisCO: Ribulose-1,5-bisphosphate carboxylase oxygenase; MMO: Methane Monooxy-genase.

## Authors' Contributions

MS (re-)implemented the entire pipeline, conducted all validation testing and wrote the manuscript. AS developed and implemented the placement algorithms and heuristics in RAxML, defined the interface to the rest of the pipeline, and helped writing the manuscript. SAB supported the systematic validations of the pipeline, validated the improvements in RAxML, and helped writing the manuscript. CVM implemented the initial versions of both the pipeline as well as the website, and wrote the manuscript.

## Additional data files

All reference information contained in MLTreeMap (sequences, phylogenies) is available from the associated website http://mltreemap.org/.

## References

[B1] Alain K Querellou J Cultivating the uncultured: limits, advances and future challenges Extremophiles 2009 13 4 583 594 10.1007/s00792-009-0261-3 19548063

[B2] Ferrari BC Winsley T Gillings M Binnerup S Cultivating previously uncultured soil bacteria using a soil substrate membrane system Nat Protoc 2008 3 8 1261 1269 10.1038/nprot.2008.102 18714294

[B3] Zengler K Central role of the cell in microbial ecology Microbiol Mol Biol Rev 2009 73 4 712 729 10.1128/MMBR.00027-09 19946138 PMC2786577

[B4] Hugenholtz P Exploring prokaryotic diversity in the genomic era Genome Biol 2002 3 2 REVIEWS0003 10.1186/gb-2002-3-2-reviews0003 11864374 PMC139013

[B5] Liolios K Chen IM Mavromatis K Tavernarakis N Hugenholtz P Markowitz VM Kyrpides NC The Genomes On Line Database (GOLD) in 2009: status of genomic and metagenomic projects and their associated metadata Nucleic Acids Res 2010 38 Database D346 354 10.1093/nar/gkp848 19914934 PMC2808860

[B6] Peterson J Garges S Giovanni M McInnes P Wang L Schloss JA Bonazzi V McEwen JE Wetterstrand KA Deal C The NIH Human Microbiome Project Genome Res 2009 19 12 2317 2323 10.1101/gr.096651.109 19819907 PMC2792171

[B7] Wu D Hugenholtz P Mavromatis K Pukall R Dalin E Ivanova NN Kunin V Goodwin L Wu M Tindall BJ A phylogeny-driven genomic encyclopaedia of Bacteria and Archaea Nature 2009 462 7276 1056 1060 10.1038/nature08656 20033048 PMC3073058

[B8] Ottesen EA Hong JW Quake SR Leadbetter JR Microfluidic digital PCR enables multigene analysis of individual environmental bacteria Science 2006 314 5804 1464 1467 10.1126/science.1131370 17138901

[B9] Zhang K Martiny AC Reppas NB Barry KW Malek J Chisholm SW Church GM Sequencing genomes from single cells by polymerase cloning Nat Biotechnol 2006 24 6 680 686 10.1038/nbt1214 16732271

[B10] Ishoey T Woyke T Stepanauskas R Novotny M Lasken RS Genomic sequencing of single microbial cells from environmental samples Curr Opin Microbiol 2008 11 3 198 204 10.1016/j.mib.2008.05.006 18550420 PMC3635501

[B11] Handelsman J Rondon MR Brady SF Clardy J Goodman RM Molecular biological access to the chemistry of unknown soil microbes: a new frontier for natural products Chem Biol 1998 5 10 R245 249 10.1016/S1074-5521(98)90108-9 9818143

[B12] Kunin V Copeland A Lapidus A Mavromatis K Hugenholtz P A bioinformatician's guide to metagenomics Microbiol Mol Biol Rev 2008 72 4 557 578 Table of Contents 10.1128/MMBR.00009-08 19052320 PMC2593568

[B13] Handelsman J Metagenomics: application of genomics to uncultured microorganisms Microbiol Mol Biol Rev 2004 68 4 669 685 10.1128/MMBR.68.4.669-685.2004 15590779 PMC539003

[B14] Eisen JA Environmental shotgun sequencing: its potential and challenges for studying the hidden world of microbes PLoS Biol 2007 5 3 e82 10.1371/journal.pbio.0050082 17355177 PMC1821061

[B15] Raes J Foerstner KU Bork P Get the most out of your metagenome: computational analysis of environmental sequence data Curr Opin Microbiol 2007 10 5 490 498 10.1016/j.mib.2007.09.001 17936679

[B16] Tringe SG Rubin EM Metagenomics: DNA sequencing of environmental samples Nat Rev Genet 2005 6 11 805 814 10.1038/nrg1709 16304596

[B17] Raes J Korbel JO Lercher MJ von Mering C Bork P Prediction of effective genome size in metagenomic samples Genome Biol 2007 8 1 R10 10.1186/gb-2007-8-1-r10 17224063 PMC1839125

[B18] Angly FE Willner D Prieto-Davo A Edwards RA Schmieder R Vega-Thurber R Antonopoulos DA Barott K Cottrell MT Desnues C The GAAS metagenomic tool and its estimations of viral and microbial average genome size in four major biomes PLoS Comput Biol 2009 5 12 e1000593 10.1371/journal.pcbi.1000593 20011103 PMC2781106

[B19] Johnson PL Slatkin M Inference of microbial recombination rates from metagenomic data PLoS Genet 2009 5 10 e1000674 10.1371/journal.pgen.1000674 19798447 PMC2745702

[B20] Tringe SG von Mering C Kobayashi A Salamov AA Chen K Chang HW Podar M Short JM Mathur EJ Detter JC Comparative metagenomics of microbial communities Science 2005 308 5721 554 557 10.1126/science.1107851 15845853

[B21] Turnbaugh PJ Hamady M Yatsunenko T Cantarel BL Duncan A Ley RE Sogin ML Jones WJ Roe BA Affourtit JP A core gut microbiome in obese and lean twins Nature 2009 457 7228 480 484 10.1038/nature07540 19043404 PMC2677729

[B22] McHardy AC Rigoutsos I What's in the mix: phylogenetic classification of metagenome sequence samples Curr Opin Microbiol 2007 10 5 499 503 10.1016/j.mib.2007.08.004 17933580

[B23] Teeling H Waldmann J Lombardot T Bauer M Glockner FO TETRA: a web-service and a stand-alone program for the analysis and comparison of tetranucleotide usage patterns in DNA sequences BMC Bioinformatics 2004 5 163 10.1186/1471-2105-5-163 15507136 PMC529438

[B24] McHardy AC Martin HG Tsirigos A Hugenholtz P Rigoutsos I Accurate phylogenetic classification of variable-length DNA fragments Nat Methods 2007 4 1 63 72 10.1038/nmeth976 17179938

[B25] Abe T Sugawara H Kinouchi M Kanaya S Ikemura T Novel phylogenetic studies of genomic sequence fragments derived from uncultured microbe mixtures in environmental and clinical samples DNA Res 2005 12 5 281 290 10.1093/dnares/dsi015 16769690

[B26] Brady A Salzberg SL Phymm and PhymmBL: metagenomic phylogenetic classification with interpolated Markov models Nat Methods 2009 6 9 673 676 10.1038/nmeth.1358 19648916 PMC2762791

[B27] Dick GJ Andersson AF Baker BJ Simmons SL Thomas BC Yelton AP Banfield JF Community-wide analysis of microbial genome sequence signatures Genome Biol 2009 10 8 R85 10.1186/gb-2009-10-8-r85 19698104 PMC2745766

[B28] Huson DH Auch AF Qi J Schuster SC MEGAN analysis of metagenomic data Genome Res 2007 17 3 377 386 10.1101/gr.5969107 17255551 PMC1800929

[B29] Krause L Diaz NN Goesmann A Kelley S Nattkemper TW Rohwer F Edwards RA Stoye J Phylogenetic classification of short environmental DNA fragments Nucleic Acids Res 2008 36 7 2230 2239 10.1093/nar/gkn038 18285365 PMC2367736

[B30] Monzoorul Haque M Ghosh TS Komanduri D Mande SS SOrt-ITEMS: Sequence orthology based approach for improved taxonomic estimation of metagenomic sequences Bioinformatics 2009 25 14 1722 1730 10.1093/bioinformatics/btp317 19439565

[B31] von Mering C Hugenholtz P Raes J Tringe SG Doerks T Jensen LJ Ward N Bork P Quantitative phylogenetic assessment of microbial communities in diverse environments Science 2007 315 5815 1126 1130 10.1126/science.1133420 17272687

[B32] Dutilh BE Snel B Ettema TJ Huynen MA Signature genes as a phylogenomic tool Mol Biol Evol 2008 25 8 1659 1667 10.1093/molbev/msn115 18492663 PMC2464742

[B33] Wu M Eisen JA A simple, fast, and accurate method of phylogenomic inference Genome Biol 2008 9 10 R151 10.1186/gb-2008-9-10-r151 18851752 PMC2760878

[B34] Schreiber F Gumrich P Daniel R Meinicke P Treephyler: fast taxonomic profiling of metagenomes Bioinformatics 2010 26 7 960 961 10.1093/bioinformatics/btq070 20172941

[B35] Felsenstein J Evolutionary trees from DNA sequences: a maximum likelihood approach J Mol Evol 1981 17 6 368 376 10.1007/BF01734359 7288891

[B36] Felsenstein J Inferring phylogenies 2004 Sunderland, Mass.: Sinauer Assoc

[B37] Whelan S Lio P Goldman N Molecular phylogenetics: state-of-the-art methods for looking into the past Trends Genet 2001 17 5 262 272 10.1016/S0168-9525(01)02272-7 11335036

[B38] Holder M Lewis PO Phylogeny estimation: traditional and Bayesian approaches Nat Rev Genet 2003 4 4 275 284 10.1038/nrg1044 12671658

[B39] Delmotte N Knief C Chaffron S Innerebner G Roschitzki B Schlapbach R von Mering C Vorholt JA Community proteogenomics reveals insights into the physiology of phyllosphere bacteria Proc Natl Acad Sci USA 2009 106 38 16428 16433 10.1073/pnas.0905240106 19805315 PMC2738620

[B40] Kunin V Raes J Harris JK Spear JR Walker JJ Ivanova N von Mering C Bebout BM Pace NR Bork P Millimeter-scale genetic gradients and community-level molecular convergence in a hypersaline microbial mat Mol Syst Biol 2008 4 198 10.1038/msb.2008.35 18523433 PMC2483411

[B41] Schmidt HA Strimmer K Vingron M von Haeseler A TREE-PUZZLE: maximum likelihood phylogenetic analysis using quartets and parallel computing Bioinformatics 2002 18 3 502 504 10.1093/bioinformatics/18.3.502 11934758

[B42] Stamatakis A RAxML-VI-HPC: maximum likelihood-based phylogenetic analyses with thousands of taxa and mixed models Bioinformatics 2006 22 21 2688 2690 10.1093/bioinformatics/btl446 16928733

[B43] Stamatakis A Hoover P Rougemont J A rapid bootstrap algorithm for the RAxML Web servers Syst Biol 2008 57 5 758 771 10.1080/10635150802429642 18853362

[B44] Birney E Clamp M Durbin R GeneWise and Genomewise Genome Res 2004 14 5 988 995 10.1101/gr.1865504 15123596 PMC479130

[B45] Durbin R Biological sequence analysis: probabilistic models of proteins and nucleic acids 1998 Cambridge [u.a.]: Cambridge Univ. Press

[B46] Talavera G Castresana J Improvement of phylogenies after removing divergent and ambiguously aligned blocks from protein sequence alignments Syst Biol 2007 56 4 564 577 10.1080/10635150701472164 17654362

[B47] Ciccarelli FD Doerks T von Mering C Creevey CJ Snel B Bork P Toward automatic reconstruction of a highly resolved tree of life Science 2006 311 5765 1283 1287 10.1126/science.1123061 16513982

[B48] Kalyuzhnaya MG Lapidus A Ivanova N Copeland AC McHardy AC Szeto E Salamov A Grigoriev IV Suciu D Levine SR High-resolution metagenomics targets specific functional types in complex microbial communities Nat Biotechnol 2008 26 9 1029 1034 10.1038/nbt.1488 18711340

[B49] Mavromatis K Ivanova N Barry K Shapiro H Goltsman E McHardy AC Rigoutsos I Salamov A Korzeniewski F Land M Use of simulated data sets to evaluate the fidelity of metagenomic processing methods Nat Methods 2007 4 6 495 500 10.1038/nmeth1043 17468765

[B50] Tringe SG Zhang T Liu X Yu Y Lee WH Yap J Yao F Suan ST Ing SK Haynes M The airborne metagenome in an indoor urban environment PLoS One 2008 3 4 e1862 10.1371/journal.pone.0001862 18382653 PMC2270337

[B51] Baker GC Smith JJ Cowan DA Review and re-analysis of domain-specific 16 S primers J Microbiol Methods 2003 55 3 541 555 10.1016/j.mimet.2003.08.009 14607398

[B52] Polz MF Cavanaugh CM Bias in template-to-product ratios in multitemplate PCR Appl Environ Microbiol 1998 64 10 3724 3730 9758791 10.1128/aem.64.10.3724-3730.1998 PMC106531

[B53] Sipos R Szekely AJ Palatinszky M Revesz S Marialigeti K Nikolausz M Effect of primer mismatch, annealing temperature and PCR cycle number on 16 S rRNA gene-targetting bacterial community analysis FEMS Microbiol Ecol 2007 60 2 341 350 10.1111/j.1574-6941.2007.00283.x 17343679

[B54] DeSantis TZ Brodie EL Moberg JP Zubieta IX Piceno YM Andersen GL High-density universal 16 S rRNA microarray analysis reveals broader diversity than typical clone library when sampling the environment Microb Ecol 2007 53 3 371 383 10.1007/s00248-006-9134-9 17334858

[B55] Wagner M Loy A Klein M Lee N Ramsing NB Stahl DA Friedrich MW Functional marker genes for identification of sulfate-reducing prokaryotes Methods Enzymol 2005 397 469 489 10.1016/S0076-6879(05)97029-8 16260310

[B56] Junier P Molina V Dorador C Hadas O Kim OS Junier T Witzel JP Imhoff JF Phylogenetic and functional marker genes to study ammonia-oxidizing microorganisms (AOM) in the environment Appl Microbiol Biotechnol 2010 85 3 425 440 10.1007/s00253-009-2228-9 19830422 PMC2802487

[B57] Braker G Zhou J Wu L Devol AH Tiedje JM Nitrite reductase genes (nirK and nirS) as functional markers to investigate diversity of denitrifying bacteria in pacific northwest marine sediment communities Appl Environ Microbiol 2000 66 5 2096 2104 10.1128/AEM.66.5.2096-2104.2000 10788387 PMC101460

[B58] Auguet JC Borrego CM Baneras L Casamayor EO Fingerprinting the genetic diversity of the biotin carboxylase gene (accC) in aquatic ecosystems as a potential marker for studies of carbon dioxide assimilation in the dark Environ Microbiol 2008 10 10 2527 2536 10.1111/j.1462-2920.2008.01677.x 18557770

[B59] Foerstner KU Doerks T Creevey CJ Doerks A Bork P A computational screen for type I polyketide synthases in metagenomics shotgun data PLoS One 2008 3 10 e3515 10.1371/journal.pone.0003515 18953415 PMC2568958

[B60] Ashida H Danchin A Yokota A Was photosynthetic RuBisCO recruited by acquisitive evolution from RuBisCO-like proteins involved in sulfur metabolism? Res Microbiol 2005 156 5-6 611 618 10.1016/j.resmic.2005.01.014 15950120

[B61] Ashida H Saito Y Kojima C Kobayashi K Ogasawara N Yokota A A functional link between RuBisCO-like protein of Bacillus and photosynthetic RuBisCO Science 2003 302 5643 286 290 10.1126/science.1086997 14551435

[B62] Imker HJ Singh J Warlick BP Tabita FR Gerlt JA Mechanistic diversity in the RuBisCO superfamily: a novel isomerization reaction catalyzed by the RuBisCO-like protein from Rhodospirillum rubrum Biochemistry 2008 47 43 11171 11173 10.1021/bi801685f 18826254 PMC2597038

[B63] Johnston AW Li Y Ogilvie L Metagenomic marine nitrogen fixation--feast or famine? Trends Microbiol 2005 13 9 416 420 10.1016/j.tim.2005.07.002 16043354

[B64] Raymond J Siefert JL Staples CR Blankenship RE The natural history of nitrogen fixation Mol Biol Evol 2004 21 3 541 554 10.1093/molbev/msh047 14694078

[B65] Ohkuma M Noda S Kudo T Phylogenetic diversity of nitrogen fixation genes in the symbiotic microbial community in the gut of diverse termites Appl Environ Microbiol 1999 65 11 4926 4934 10543805 10.1128/aem.65.11.4926-4934.1999 PMC91663

[B66] Jensen LJ Kuhn M Stark M Chaffron S Creevey C Muller J Doerks T Julien P Roth A Simonovic M STRING 8--a global view on proteins and their functional interactions in 630 organisms Nucleic Acids Res 2009 37 Database D412 416 10.1093/nar/gkn760 18940858 PMC2686466

[B67] Pruitt KD Tatusova T Klimke W Maglott DR NCBI Reference Sequences: current status, policy and new initiatives Nucleic Acids Res 2009 37 Database D32 36 10.1093/nar/gkn721 18927115 PMC2686572

[B68] Kanehisa M Araki M Goto S Hattori M Hirakawa M Itoh M Katayama T Kawashima S Okuda S Tokimatsu T KEGG for linking genomes to life and the environment Nucleic Acids Res 2008 36 Database D480 484 18077471 10.1093/nar/gkm882 PMC2238879

[B69] Markowitz VM Ivanova NN Szeto E Palaniappan K Chu K Dalevi D Chen IM Grechkin Y Dubchak I Anderson I IMG/M: a data management and analysis system for metagenomes Nucleic Acids Res 2008 36 Database D534 538 17932063 10.1093/nar/gkm869 PMC2238950

[B70] Seshadri R Kravitz SA Smarr L Gilna P Frazier M CAMERA: a community resource for metagenomics PLoS Biol 2007 5 3 e75 10.1371/journal.pbio.0050075 17355175 PMC1821059

